# Interpretable deep learning for depression detection in neurological patients using EEG signals

**DOI:** 10.1016/j.mex.2025.103736

**Published:** 2025-11-28

**Authors:** Parisa Khaleghi, Duygu Cakir, Ali Hamidoğlu, Omer Melih Gul, Seifedine Kadry

**Affiliations:** a Department of Artificial Intelligence, Faculty of Engineering and Natural Sciences, Bahçeşehir University, Turkey; b Software Engineering, Faculty of Engineering and Natural Sciences, Bahçeşehir University, Turkey; c Interdisciplinary Lab for Mathematical Ecology and Epidemiology (ILMEE) & Department of Mathematical and Statistical Sciences, University of Alberta, Edmonton, Alberta, Canada; d Department of Mathematics, Faculty of Engineering and Natural Sciences, Bahçeşehir University, Turkey; e Informatics Institute, Istanbul Technical University 34469, Sariyer, Istanbul, Turkey; f Department of Computer Engineering, Bahçeşehir University 34353, Besiktas, Istanbul, Turkey; g Department of Computer Science and Mathematics, Lebanese American University, Beirut, Lebanon

**Keywords:** Electroencephalography, Depression detection, Explainable artificial intelligence, SHAP analysis, EEG biomarkers

## Abstract

Depression affects over 280 million people worldwide, with neurological patients particularly prone to medication-induced episodes. Conventional diagnostic approaches rely on subjective evaluations, limiting reproducibility and consistency in clinical settings. This study proposes an interpretable deep learning framework for objective depression detection using EEG signals. We hypothesize that combining EEG-based features with explainable artificial intelligence can provide both high accuracy and transparency in diagnosis. The model was trained on EEG data from 232 neurological patients, achieving 98 % classification accuracy. Interpretability was enhanced through SHAP (SHapley Additive exPlanations) analysis, which identified clinically meaningful EEG biomarkers such as the delta/alpha ratio and theta band power. This paper highlights the following contributions:

Integration of EEG features with a lightweight deep learning model for depression detection

High diagnostic accuracy achieved while maintaining interpretability for clinicians

An objective tool that is compatible with existing EEG infrastructure, supporting clinical adoption

These results show that our framework bridges predictive performance with interpretability, offering a transparent and scalable EEG-based diagnostic tool. We conclude that this approach can complement clinical decision-making, reducing dependence on subjective evaluation and enabling more consistent, data-driven mental health care.

## Graphical abstract

**Figure:**
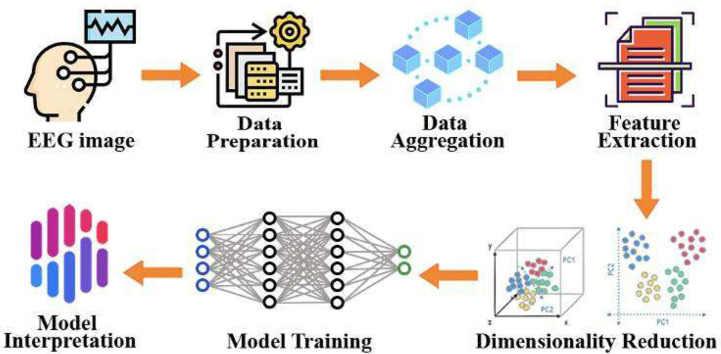

## Specifications table

**Table:** 

Subject area	Bioinformatics
**More specific subject area**	Deep learning and SHAP-based interpretation of EEG for depression detection
**Name of your method**	Interpretable Deep Learning Framework for EEG-based Depression Detection
**Name and reference of original method**	Lundberg, S.M., & Lee, S.I. (2017). A unified approach to interpreting model predictions. Advances in Neural Information Processing Systems, 30 (NeurIPS) [SHAP reference]
**Resource availability**	Tocodeforsoul, “Depression rest EEG features,” 2023, Accessed: September 30, 2024 [Online]. Available: https://www.kaggle.com/datasets/tocodeforsoul/depression-rest-eeg-features

## Background

Depression is one of the most prevalent mental health disorders worldwide, affecting approximately 280 million individuals [1] and constituting a leading cause of disability [2,3]. Its burden is particularly acute in patients with neurological disorders, who face disproportionately high risks of developing depressive episodes. In this population, depression arises from a multifaceted interplay of factors: the neurobiological impact of the primary disorder, psychosocial consequences of chronic illness, and adverse effects of neurological medications on brain chemistry [4,5]. For example, certain antiepileptic drugs such as barbiturates induce depressive symptoms in roughly 10 % of patients, while levetiracetam has been linked to depression in up to 22 % of patients [5]. This reciprocal relationship can increase the possibility for vulnerability to depression, which makes it necessary to have an accurate, early detection [6,7].

Traditional approaches for detecting depression depend on clinical interviews, tests, self-report data, and behavioral analysis. There are several drawbacks and limitations when applying traditional ways, such as wrongly taken self-reports, personal data, or overlapping neurological and psychiatric symptoms [8]. Hence, those approaches often lack reproducibility and may fail to observe the true case regarding the depression.

A recent approach, Electroencephalography (EEG), has been offering a promising modality for objective depression detection. In this context, EEG signals are considered non-invasive and routinely used in neurological practice, and they are capable of capturing neural activity at millisecond temporal resolution [9,10]. On the other hand, adding it to early depression detection may not be too much work.

Using EEG signals in biomedical signal analysis, deep learning (DL) has shown a promising method for early disease detection [11,12],. Here, convolutional neural networks (CNNs) prove themselves to capture complex temporal and spatial patterns in EEG data, enabling neurological feature learning through training clinical datasets [13,14]. Although CNNs have high predictive power, they are often considered as “black-box” models, which make them costly to implement, especially when healthcare professionals want transparency to trust predictions provided by them [[15], [16], [17]]. Even very accurate models from CNNs have trouble working with clinical workflows if they can't be understood completely, which makes them less useful and applicable in neurological disease detections.

Motivated by this, we propose SHapley Additive Explanations (SHAP), an explainable AI technique to address these challenges by illustrating each input feature by means of its marginal contribution to the respective disease [18,19]. In this framework, SHAP analysis uses EEG-based signals and transforms them into explainable platforms by using delta/alpha ratios or theta power and useful markers.

Here, our proposed model integrates multi-domain EEG feature extraction, dimensionality reduction techniques, and CNN-based classification. Such integration provides a rich feature extraction as well as outstanding computational efficiency. We aim to create a useful, interpretable, and clinically understandable tool for early detection of depression by combining high accuracy with clear decision pathways. The design uses existing EEG technology, which means that it won't interfere with normal neurological tests. It could also make it easier to intervene early, encourage patients to stick to their treatment plan, and improve overall treatment outcomes.

## Methodology

This section explains how to use EEG signals to find depression in neurological patients using DL. The method combines advanced signal processing techniques, dimensionality reduction methods, CNNs classification, and explainable AI to get high diagnostic accuracy and clinical interpretability.

### Dataset and participants

The study uses the publicly accessible Depression-Rest EEG dataset [20] from Kaggle, including EEG recordings from 232 patients, meticulously balanced to feature an equal distribution of depressed (*n* = 116) and non-depressed (*n* = 116) individuals, previously gathered and clinically classified. The dataset is put together to include patients with diverse neurological conditions (epilepsy, multiple sclerosis, movement disorders), representing a clinically relevant neurological patient population. The original dataset does not explain which psychometric tools are used to diagnose depression or if structured clinical interviews are done. However, the dataset does have reliable clinical labels that we use to build our model.

Participants are chosen from neurological patient populations to ensure clinical relevance to the intended application domain. The dataset includes patients with a range of neurological disorders, including epilepsy, multiple sclerosis, and movement disorders.

This study uses a publicly accessible dataset rather than performing diagnoses as part of our research. In this context, EEG recordings identify 31 unique features within six cerebral regions: frontal, central, temporal, parietal, occipital, and prefrontal areas. The full set of features included traditional biomarkers like mobility, complexity, delta/alpha ratio, FFT theta max power, and wavelet detailed entropy, as well as statistical measures like mean, standard deviation, skewness, and kurtosis.

The EEG feature extraction process generates a comprehensive set of features across multiple domains (statistical, frequency-domain, and time-frequency). Table 1 presents the complete set of 31 features extracted for this analysis. Features are organized by extraction method:

- Statistical Features: Mobility, Complexity, Min, Max, STD, Mean, Median, Activity, Kurtosis, Skewness (10 features)
- Frequency Domain Features: Delta/Alpha Ratio, FFT Theta Max Power, FFT Delta Max Power, FFT Alpha Max Power, FFT Beta Max Power, 1st Difference Max, 2nd Difference Max, 1st Difference Mean, 2nd Difference Mean, Coefficient of Variation (10 features)
- Wavelet Features: Wavelet Detailed Entropy, Wavelet Approximate Mean (2 features)
- Additional Features (computed across regions): Additional derived features and cross-regional statistics (9 features)

**Table 1:** Complete set of 31 EEG features extracted for depression classification analysis. Features span statistical, frequency-domain, and time-frequency domains.

DOMAIN	FEATURE NAME	TYPICAL RANGE	DEPRESSION RANGE
STATISTICAL	Mobility	0.30–0.40	< 0.30
STATISTICAL	Complexity	4.0–5.0	< 4.0
STATISTICAL	Min	-	-
STATISTICAL	Max	-	-
STATISTICAL	STD (Standard Deviation)	-	-
STATISTICAL	Mean	-	-
STATISTICAL	Median	-	-
STATISTICAL	Activity	-	-
STATISTICAL	Kurtosis	-	-
STATISTICAL	Skewness	-	-
FREQUENCY	Delta/Alpha Ratio	50–70	> 70
FREQUENCY	FFT Theta Max Power	0.0005–0.0007	> 0.0007
FREQUENCY	FFT Delta Max Power	-	-
FREQUENCY	FFT Alpha Max Power	-	-
FREQUENCY	FFT Beta Max Power	-	-
FREQUENCY	1st Difference Max	-	-
FREQUENCY	2nd Difference Max	-	-
FREQUENCY	1st Difference Mean	-	-
FREQUENCY	2nd Difference Mean	-	-
FREQUENCY	Coefficient of Variation	-	-
TIME-FREQ	Wavelet Detailed Entropy	0.0012–0.0015	< 0.0012
TIME-FREQ	Wavelet Approximate Mean	-	-
DERIVED	Theta Band Power (avg)	-	-
DERIVED	Alpha Band Power (avg)	-	-
DERIVED	Delta Band Power (avg)	-	-
DERIVED	Beta Band Power (avg)	-	-
DERIVED	Hjorth Activity	-	-
DERIVED	Signal Complexity (spectral)	-	-
DERIVED	Regional Power Asymmetry	-	-

The EEG features selected for analysis and their corresponding thresholds distinguishing between depressed and healthy subjects are summarized in Table 2. The EEG feature thresholds for identifying depression listed in the table, such as increased theta power, elevated delta-alpha ratio, reduced wavelet entropy, and decreased Hjorth mobility and complexity, are consistent with state-of-the-art EEG-based depression studies indicating characteristic slowing and reduced signal complexity in depressed patients [[21], [22], [23]].

**Table 2:** Depression-related EEG features and their normal versus abnormal threshold ranges.

Feature	Normal Range	Depression Range
FFT Theta Max Power	0.0005–0.0007	*>* 0*.*0007
Wavelet Detailed Entropy	0.0012–0.0015	*<* 0*.*0012
Mobility	0.30–0.40	*<* 0*.*30
Complexity	4.0–5.0	*<* 4*.*0
Delta/Alpha Ratio	50–70	*>* 70

Normal ranges for clinical depression are well-established only for the 5 primary biomarkers (Table 1, Table 2). The other 26 features are complementary measures that help the model distinguish between groups, but they don't have published 'depression ranges' in the literature because they're feature-specific. This is why we used machine learning rather than threshold-based rules in which the model learns which feature combinations are most discriminative.

The employed dataset provides binary depression classifications assigned by the original investigators; however, the dataset documentation does not specify which standardized depression rating scales, if any, were used in diagnosis. Moreover, additional clinical characterization variables such as age, sex, neurological diagnoses, comorbidities, and medication status were not available in the employed public dataset.

### Feature extraction pipeline

The feature extraction pipeline employed multiple complementary techniques to capture comprehensive EEG signal characteristics across time, frequency, and statistical domains. Each method was selected based on its documented utility in EEG analysis and depression research. Table 3 shows key parameters in Wavelet transform analysis using the Daubechies-4 (db4) wavelet to decompose EEG signals into multiple frequency scales.

**Table 3:** EEG frequency bands and their associated neurophysiological states relevant to depression analysis.

Frequency Band	Range (Hz)	Associated States
Delta	0–4	Deep sleep, unconscious states
Theta	4–8	Drowsiness, meditation, early sleep
Alpha	8–13	Relaxed, calm states
Beta	13–30	Active thinking, problem-solving
Gamma	30–50	High-level cognitive processing

*Wavelet Transform Analysis:* The db4 wavelet offers time-frequency localization with an acceptable compromise between temporal accuracy and frequency resolution [21,22].

*Power Spectral Density Analysis:* We use Welch's method to measure how energy is spread out across standard EEG frequency bands. Welch's method enhances spectral estimates relative to the standard FFT by diminishing variance through averaging [28]. The analysis concentrates on five frequency bands, including delta (0–4 Hz), theta (4–8 Hz), alpha (8–13 Hz), beta (13–30 Hz), and gamma (30–50 Hz). Theta and alpha bands receive particular attention in depression research due to documented alterations in depressed populations: elevated theta power and reduced alpha power have been consistently reported [21,22]. The 256 Hz sampling frequency and associated parameter selection followed standard EEG protocols.

Short-Time Fourier Transform (STFT): STFT offers time-frequency representations that record changes over time in spectral content [24]. In biomedical signal processing, STFT is often used to determine parts that are not stationary. This technique identifies dynamic fluctuations in neural oscillations that may be different in EEG recording sessions. The parameters (sampling frequency 256 Hz, segment length 128 samples) are chosen to strike a balance between time and frequency resolution.

Statistical Feature Extraction: For each EEG signal, we find the mean, standard deviation, skewness, and kurtosis. These measures describe the properties of signal distribution, showing neurons working. Previous studies have shown that depressed patients' EEG signals have higher skewness and kurtosis [22]. We calculated the Hjorth parameters (activity, mobility, and complexity) because they measure EEG complexity, which is lower in people with depression.

Data Aggregation: Based on depression diagnosis, aggregation rules are used on the 31 features taken from six brain areas. We combined the maximum values of the delta/alpha ratio and the FFT theta max power across regions to determine features that are likely to show depression when they are high. We combine features (mobility, complexity, and wavelet detailed entropy) that exhibit depression signs when their values are at their lowest. The remaining features are combined using mean values to create stable, central tendency representations.

Integration of statistical, frequency-domain, and time-frequency features has been shown to make it easier to detect depression than using only one type of feature [9,22]. By merging data from different time, frequency, and complexity domains, the feature set captures a wide range of changes in EEG that are linked to depression.

### Dimensionality reduction

We use a two-step process to reduce the number of dimensions. First, we use principal component analysis (PCA) [25] to reduce the number of linear dimensions, and then we use t-distributed stochastic neighbor embedding (t-SNE) [26] to observe and group the data in a nonlinear way. This combination takes advantage of the strengths of both methods, especially when EEG feature spaces are very high-dimensional.

Principal component analysis is set up to keep the first 21 components from the standardized comprehensive feature matrix. This keeps about 95 % of the total variance in the original feature space. The choice of 21 components provides a balance between keeping information and making calculations faster. This methodology makes sure that important signal characteristics are kept and noise and redundancy are prevented, which eventually lowers the risk of the curse of dimensionality.

To attain stable low-dimensional representations of the PCA-transformed data, t-SNE visualization uses a perplexity of 50 and 300 iterations. The PCA-tSNE method works well for performing class separability and getting data ready for training CNN.

### CNN architecture

The CNN architecture is designed according to EEG-based depression classification, using layers to optimize temporal signal processing and regularization techniques to prevent overfitting.

**Fig. 2:**
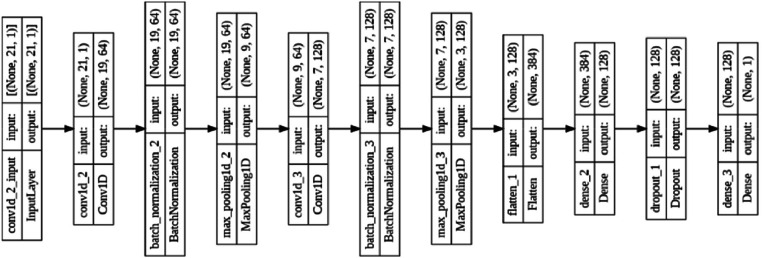
Detailed CNN architecture showing the sequential layers from input through convolutional processing, pooling, and dense classification layers with specified parameters for each component.

Fig. 2 shows the full structure of the lightweight network, where the input goes through convolutional layers and ends up as a classification output. The network starts with a one-dimensional convolutional layer that uses 64 filters with a kernel size of 3. After each convolutional layer, batch normalization is used to make training more stable and speed convergence.

A second convolutional layer uses 128 filters with a kernel size of 3 to discover higher-level feature combinations and relationships over time. The higher number of filters makes it possible to learn more complicated pattern hierarchies, and the constant kernel size keeps the temporal resolution right for EEG signal characteristics.

The network architecture has a flattening layer that shifts from convolutional feature maps to fully connected processing. After that, there would be a dense layer with 128 neurons and ReLU activation. This fully connected layer provided capacity for learning complex nonlinear relationships between extracted features and depression classifications.

Dropout regularization with 50 % probability was applied to prevent overfitting, randomly deactivating neurons during training to improve generalization. The final output layer utilized a single neuron with sigmoid activation for binary classification, providing probability estimates for depression presence. The complete architecture specifications are detailed in Table 4.

**Table 4:** CNN architecture specifications including layer types, parameters, and functional descriptions.

Layer	Parameters	Description
Conv1D	64 filters, kernel=3	Feature extraction
BatchNorm	—	Training stabilization
MaxPool1D	pool size=2	Dimensionality reduction
Conv1D	128 filters, kernel=3	Higher-level features
BatchNorm	—	Training stabilization
MaxPool1D	pool size=2	Dimensionality reduction
Flatten	—	Feature map conversion
Dense	128 neurons, ReLU	Nonlinear mapping
Dropout	rate=0.5	Overfitting prevention
Dense	1 neuron, sigmoid	Binary classification

Training procedures employed Adam optimization with learning rate 0.0001, selected through systematic hy- per parameter tuning. Binary cross-entropy loss function was utilized for optimization, appropriate for the binary classification task. Early stopping with patience of 10 epochs prevented overfitting while learning rate reduction with factor 0.2 provided adaptive optimization when validation performance plateaued.

The training includes 100 maximum epochs with batch size 64, balancing computational efficiency with gradient estimation quality. Class weights are computed to address any remaining class imbalance, ensuring equal contribution from both depression and non-depression samples during training. Validation processes use stratified train-test splitting with a 70–30 ratio. The stratification keeps the same number of depressed and non-depressed patients in each data split. Cross-validation using 5-fold stratified procedures provides robust performance estimates and ensures that all data contributed to both training and validation phases.

### Model interpretability

SHAP analysis provides model interpretability by quantifying the contribution of each input feature to individual predictions. Using a subset of training data as background samples, Kernel SHAP is implemented to generate explanations for the trained CNN model. The kernel approach enabled explanation of complex DL models by approximating SHAP values through local linear models.

SHAP values and PCA component loadings are utilized together in feature importance calculations to link explanations back to the original EEG features. The methodology identifies the original EEG characteristics that have the greatest impact on depression classifications.

## Method validation

This section presents the validation of our SHAP-based CNN approach for EEG-based depression detection.

### Dimensionality reduction outcomes

Principal component analysis reduces the high-dimensional EEG feature space and preserves 95.2 % of the total variance using 21 components. The first three principal components account for 34.7 %, 18.9 %, and 12.4 % of explained variance, respectively, which indicates substantial information concentration in the leading components.

**Fig. 3:**
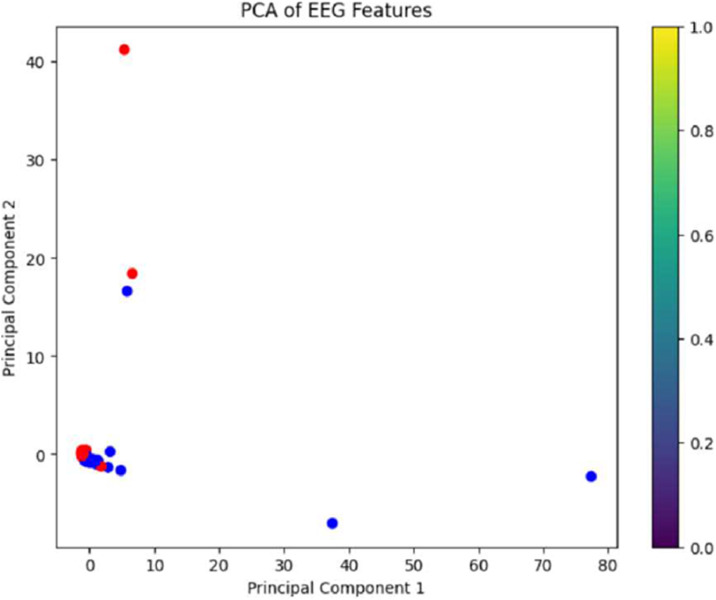
PCA visualization showing the first two principal components with limited separation between depression (red) and non-depression (blue) classes, demonstrating the need for additional dimensionality reduction techniques.

**Fig. 4:**
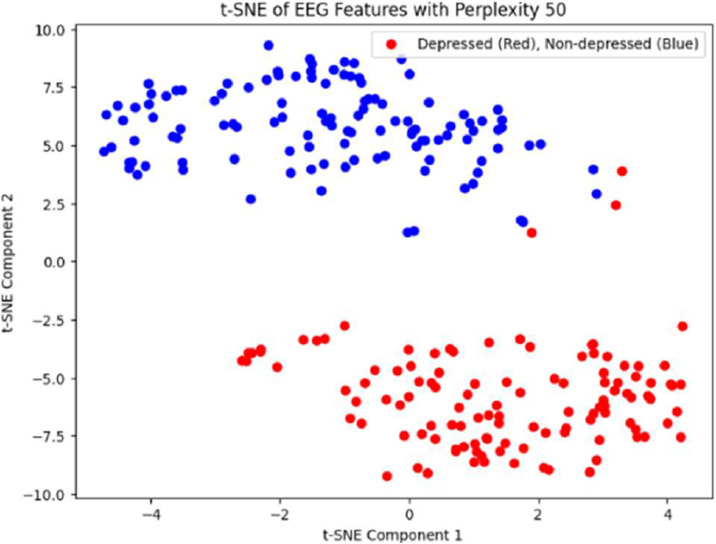
t-SNE visualization demonstrating clear separation between depression (red) and non-depression (blue) classes after nonlinear dimensionality reduction, indicating improved discriminative capacity.

Fig. 3 shows that there is a moderate separation between depression and non-depression classes. On the other hand, Fig. 4 illustrates that the t-SNE analysis, with a perplexity of 50 and 300 iterations, significantly improves class separability in comparison to PCA alone.

### CNN performance

**Fig. 5:**
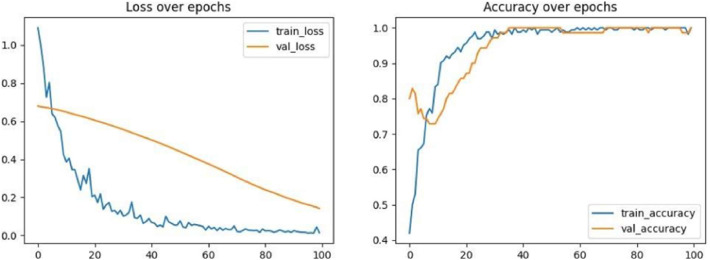
CNN training and validation performance curves showing loss reduction (left) and accuracy improvement (right) over training epochs.

The CNN achieves 98.0 % accuracy on the held-out test set on a 5-fold stratified validation as illustrated in Fig. 5. Detailed classification metrics revealed balanced performance across both depression and non-depression classes. Precision scores of 0.97 for non-depressed and 0.98 for depressed patients indicated minimal false positive rates. Recall values of 0.98 for non-depressed and 0.97 for depressed patients demonstrated effective identification of true positives in both classes. F_1_-scores of 0.98 for both classes confirmed optimal balance between precision and recall, as summarized in Table 5.

**Table 5:** Detailed classification performance metrics showing precision, recall, F_1_-score, and support for each class.

Class	Precision	Recall	F_1_-Score	Support
Non-depressed (0)	0.97	0.98	0.98	35
Depressed (1)	0.98	0.97	0.98	35
Accuracy		0.98		70
Macro Avg	0.98	0.98	0.98	70
Weighted Avg	0.98	0.98	0.98	70

Cross-validation results demonstrated robust performance across different data subsets, with mean accuracy of 97.6 % ± 1.2 % and minimal variation between folds. The consistent performance across validation folds indicated good generalizability and stability of the proposed approach.

To the best of our knowledge, very few studies have utilized the Kaggle “Depression Rest EEG Features” dataset (tocodeforsoul) for EEG-based depression detection tasks. As shown in Table 6 only the recent work by Abidi et al [27] has explicitly reported results on this dataset, achieving an accuracy of 96.0 %. Our proposed method surpasses this performance, obtaining an accuracy and F_1_-score of 98.0 %. The scarcity of research utilizing this publicly available dataset underscores the novelty and importance of the present study in providing a robust baseline and encouraging further research and comparative analyses using the same benchmark.

**Table 6:** Comparison of the proposed method with recent state-of-the-art EEG-based depression detection approaches.

Method	Year	Accuracy ( %)	F_1_-Score ( %)
EEGDepressionNet (SA-Gated DenseNet + COIWSO) [27]	2024	96.0	–
Proposed Method (Light-weight CNN + SHAP)	2025	98.0	98.0

### Model interpretability analysis

**Fig. 6:**
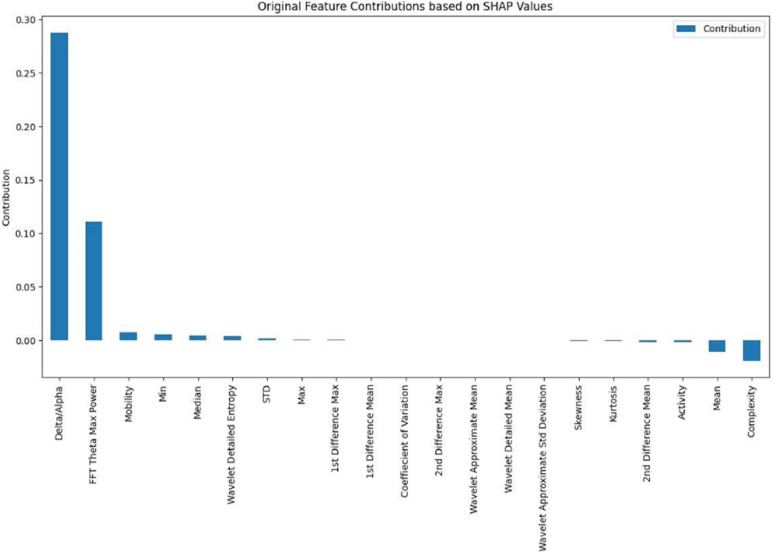
SHAP summary plot showing feature importance rankings with delta/alpha ratio and FFT theta max power as the most influential features (red indicates positive impact, blue indicates negative impact on depression classification).

**Fig. 7:**
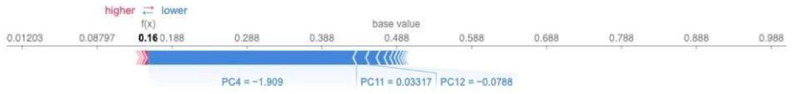
SHAP force plot for an individual prediction showing how specific principal components push the prediction toward or away from depression classification, with PC4 showing the strongest negative influence.

SHAP analysis identifies the delta/alpha ratio and FFT theta max power as illustrated in Fig. 6. Moreover, the feature importance hierarchy reveals nine features with substantial positive influence on depression classification, six features with moderate influence, and six features with minimal contribution. Moreover, principal component 4 has the biggest negative effect on predictions of depression, while components 11 and 3 have smaller positive effects. These patterns are consistent across several individual predictions, showing that the model is stable. Fig. 7 shows a typical example of how to explain a single prediction.

### Numerical validation

Numerical validation is performed by using four representative test cases including 2 depressed and 2 non-depressed patients. The test cases included two non-depressed patients with normal EEG feature ranges and two depressed patients exhibiting characteristic biomarker patterns. Table 7 presents the detailed feature values and classification results for these representative cases.

**Table 7:** Sample test cases showing EEG feature values for representative patients (P1-P4) from both depression and non-depression groups.

Feature	P1	P2	P3	P4
Classification Results				
Actual Class	No	No	Yes	Yes
Predicted Class	No	No	Yes	Yes
Confidence	0.41	0.43	0.59	0.57
EEG Features				
Delta/Alpha	0.30	0.25	−0.25	−0.30
FFT Theta	−0.40	−0.35	0.50	0.45
Mobility	−0.20	−0.10	0.40	0.50
Complexity	0.10	0.15	−0.10	−0.15

**Fig. 8:**
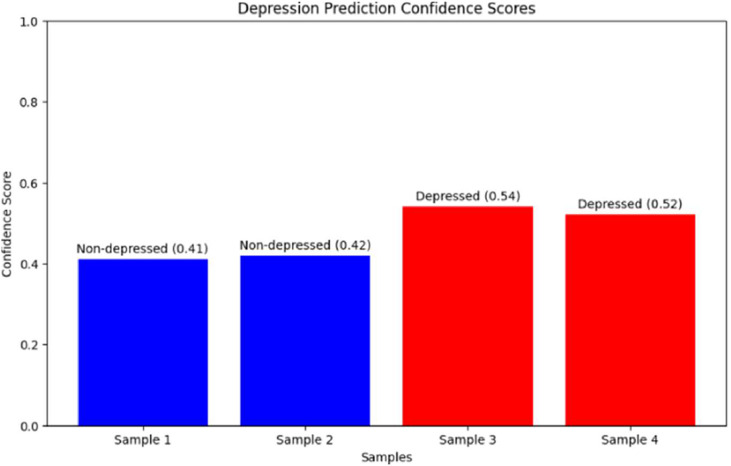
Confidence score distribution for the four test cases showing correct classification with moderate confidence levels ranging from 0.41 to 0.59.

Fig. 8 shows the confidence scores ranging from 0.41 to 0.59 across test cases. Error analysis of the entire test set finds two cases are incorrectly classified out of 70 total predictions. Both errors involve patients with borderline feature values close to classification thresholds, which shows that classification uncertainty predominantly arises in ambiguous situations rather than definitive cases.

## Limitations

There are several limitations of the proposed methodology.

First, the study uses a dataset size of 232 patients, which remains modest for DL applications. Although the size is considered substantial for EEG-based depression studies, it makes it difficult to generalize findings across broader and more heterogeneous neurological populations.

The second is regarding the confidence scores for the proposed model. These scores are moderate, even though the classification accuracy is high. Such an outcome could mean that the model needs to be calibrated better. In other words, the gap between accuracy and prediction certainty could mean that the model is overfitting to the training data or not using calibration procedures enough.

Finally, the study lacks correlation between model predictions and standardized clinical depression rating scales. The publicly available EEG dataset employed in this research included only binary depression classifications (depressed/non-depressed) and lacked continuous severity scores from validated instruments. To enhance clinical applicability, future research should validate model performance on datasets that include both EEG recordings and standardized depression assessment scores. Such data would allow for correlation analyses between model confidence and clinical severity, support the definition of optimal decision thresholds for different clinical contexts, and enable regression-based modelling of continuous severity scores for finer clinical interpretation. Furthermore, access to psychometrically validated instruments would allow comparison of model predictions against established state-of-the-art measures. While the present study establishes a methodological foundation demonstrating the feasibility of EEG-based machine learning for binary depression classification, future validation against standardized clinical scales remains essential for clinical translation.

## Ethics statements

This study did not involve any experiments on humans or animals. The analysis was conducted entirely on an existing, publicly available EEG dataset that had been previously collected with appropriate ethical approval by the original investigators. No new data were gathered, and no identifiable personal information was used in this research.

## CRediT authorship contribution statement

**Parisa Khaleghi:** Conceptualization, Formal analysis, Investigation, Methodology, Validation, Software, Visualization, Data curation, Writing – original draft. **Duygu Cakir:** Methodology, Validation, Data curation, Writing – original draft. **Ali Hamidoğlu:** Conceptualization, Methodology, Validation, Visualization, Supervision, Writing – original draft, Writing – review & editing. **Omer Melih Gul:** Methodology, Validation, Supervision, Writing – original draft. **Seifedine Kadry:** Validation, Writing – review & editing.

## Declaration of competing interest

The authors declare that they have no known competing financial interests or personal relationships that could have appeared to influence the work reported in this paper.

## Data Availability

Data will be made available on request.
